# Reference Values for DXA-Derived Visceral Adipose Tissue in Adults 40 Years and Older from a European Population: The Tromsø Study 2015–2016

**DOI:** 10.1155/2021/6634536

**Published:** 2021-05-15

**Authors:** Marie W. Lundblad, Bjarne K. Jacobsen, Jonas Johansson, Emanuella De Lucia Rolfe, Sameline Grimsgaard, Laila A. Hopstock

**Affiliations:** ^1^Department of Community Medicine, UiT The Arctic University of Norway, Tromsø, Norway; ^2^NIHR Cambridge Biomedical Research Centre—Diet, Anthropometry and Physical Activity Group, MRC Epidemiology Unit, Institute of Metabolic Science, University of Cambridge, Cambridge CB2 0QQ, UK

## Abstract

**Background:**

Reference values for visceral adipose tissue (VAT) are needed and it has been advocated that body composition measures depend on both the technique and methods applied, as well as the population of interest. We aimed to develop reference values for VAT in absolute grams (VATg), percent (VAT%), and as a kilogram-per-meters-squared index (VATindex) for women and men, and investigate potential differences between these measures and their associations with cardiometabolic risk factors (including metabolic syndrome (MetS)).

**Methods:**

In the seventh survey of the population-based Tromsø Study, 3675 participants (aged 40–84, 59% women) attended whole-body DXA scans (Lunar Prodigy GE) from where VAT was derived. We used descriptive analysis, correlations, receiver operating characteristics (ROC), and logistic regression to propose reference values for VAT and investigated VAT's association with cardiometabolic risk factors, MetS and single MetS components. Further, Youden's index was used to suggest threshold values for VAT.

**Results:**

VATg and VATindex increased until age 70 and then decreased, while VAT% increased with age across all age groups. VAT (all measurement units) was moderate to highly correlated and significantly associated with all cardiometabolic risk factors, except for total cholesterol. Associations between MetS, single MetS components, and VATg and VATindex were similar, and VAT% did not contribute any further to this association.

**Conclusion:**

These VAT reference values and thresholds, developed in a sample of adults of Norwegian origin, could be applied to other studies with similar populations using the same DXA device and protocols. The associations between VAT and cardiometabolic risk factors were similar across different measurement units of VAT.

## 1. Introduction

The definition of overweight and obesity is “abnormal or excessive fat accumulation that may impair health” [1]. The most frequently used definitions of overweight and obesity are based on anthropometric measures such as body mass index (BMI) and waist circumference, which do not distinguish between fat mass and fat free mass, but rather represent overall body size. On the contrary, available body composition measures directly address the definition of overweight and obesity. There are several tools to determine body composition. Dual energy X-ray absorptiometry (DXA) determines body composition from scanning the body with X-ray beams that pass through the body and establish amount of fat mass, bone mass, and soft tissue lean mass based on composition of the tissues [2–4]. Visceral adipose tissue (VAT) located intra-abdominally (behind the abdominal muscles and around organs) is more metabolically active than subcutaneous adipose tissue and has been associated with insulin resistance, the metabolic syndrome (MetS), cardiovascular disease, and several types of cancer [5]. DXA provides area-specific body composition, and VAT is estimated from fat mass located in the abdominal area when subcutaneous fat has been removed [3]. DXA-derived VAT has been validated against both MRI and CT [3, 6].

There are no generally accepted reference values for VAT. Previous studies have presented normative data, and there is a need for age-, sex-, and ethnicity-specific reference values [7–9]. In addition, different types of DXA systems and models as well as the type of unit used challenge VAT comparisons across studies. The most commonly parameters reported from the DXA systems are VAT mass expressed in grams (VATg) and VAT volume expressed in cubic centimeters (VATcm^3^). There is a strong positive correlation between height and weight, and likely also between height and VAT, and between body fat and VAT. To increase comparability between individuals, it is of interest to adjust for potential confounding effects of height and central adiposity, warranting relative VAT values like VATindex (VAT/height^2^) and percent visceral fat in the abdominal area (VAT%). Previous studies developing VAT reference values have highlighted the importance of technique and population-specific reference values [7]. Most other studies have used iDXA models [8, 10] or only included young adults [9, 11]. In addition, few studies have investigated the association between VAT and cardiometabolic risk factors [7, 9, 11, 12].

We aimed to develop reference values for DXA-derived VAT expressed in absolute and relative terms in an adult population, predominantly of European origin from the Tromsø Study, Norway. Additionally, we have investigated the associations of distinct VAT parameters with cardiometabolic risk factors, MetS and single MetS components. Further, we present suggested threshold values of VAT based on ability to predict MetS.

## 2. Materials and Methods

### 2.1. Study Population

**Figure 1:**
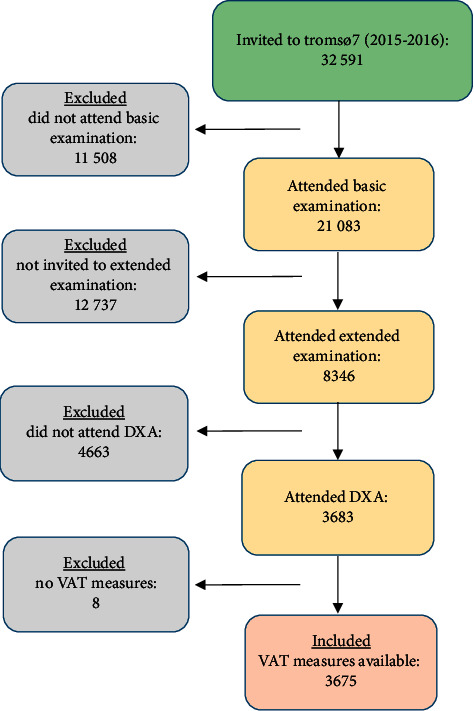
Inclusion of participants.

The Tromsø Study is an ongoing population-based study [13] consisting of seven surveys (Tromsø 1–7) conducted from 1974 to 2016, inviting large representative samples of the population in the Tromsø municipality in Northern Norway. We included participants from Tromsø 7 (2015–2016) where all inhabitants aged 40 years and older (*N* = 32591) were invited to a basic examination including questionnaires, clinical measurements, and biological sampling (Figure 1). A subsample (*N* = 13028) was premarked for invitation to extended examinations approximately two weeks later. This subsample consisted of a randomized sample (*N* = 9925) as well as previous participants attending DXA, echocardiogram, and eye examinations in Tromsø 6 (2007–2008) (*N* = 3103). A total of 21083 women and men aged 40–99 years attended the basic examination (65%), and 8346 attended the extended examinations (of the premarked sample; 64% of the originally premarked and 90% of those attending the basic examination). Of these, 3683 participated in DXA scans from whom 3675 had VAT measures available (Figure 1).

This project was approved by the Regional Committee for Medical Research Ethics (REC North ref. 2017/1967), and all participants gave written informed consent.

### 2.2. Study Measures

All measurements were performed by trained staff using standard protocols.

#### 2.2.1. Cardiometabolic Risk Factors

We included information on diabetes and use of medications from self-administered questionnaires. Nonfasting blood samples were analyzed for total cholesterol (mmol/L), high-density lipoprotein (HDL) cholesterol (mmol/L), triglycerides (mmol/L), and glycated hemoglobin (HbA1c) (%), at the Department of Laboratory Medicine at the University Hospital of North Norway (ISO certification NS-EN ISO 15189 : 2012). Blood pressure was measured three times with two-minute intervals with a Dinamap ProCare 300 monitor (GE Healthcare, Norway) and the mean of the two last readings was used in the analysis. We used MetS components based on the NCEP ATP III diagnostic criteria for the MetS (2005 revision): hypertension (mean systolic blood pressure >130 mmHg and/or mean diastolic blood pressure >85 mmHg and/or use of antihypertensives), high nonfasting blood lipids (triglycerides ≥1.7 mmol/L and/or use of lipid-lowering drugs), low HDL cholesterol (HDL cholesterol <1.3 (women) or <1.0 (men) mmol/L and/or use of lipid-lowering drugs), and diabetes (self-reported diabetes and/or HbA1c ≥ 6.5% and/or use of insulin and/or other diabetes medication) [14]. As investigation of VAT was our main objective, we excluded elevated waist circumference as a criterion. MetS was defined as presence of three or more of the MetS components presented above, as defined by NCEP ATP III (*n*: 493 (24%) women and 406 (28%) men).

#### 2.2.2. Adiposity Measures

Weight and height were measured with light clothing and no shoes to the nearest 0.1 kilograms (kg) and 0.1 centimeters (cm) using the Jenix DS-102 height and weight scale (Dong Sahn Jenix, Seoul, Korea). Waist and hip circumference were measured to the nearest 0.1 cm with a Seca measurement tape at the level of the umbilicus and the greater trochanters, respectively. BMI (weight in kg divided by height in meters (m) squared) was defined as normal (<25 kg/m^2^), overweight (25–29.9 kg/m^2^), or obese (≥30 kg/m^2^). The 31 women and 2 men with underweight (BMI <18.5 kg/m^2^) were merged with the normal weight category.

Whole-body DXA scans (Lunar GE Prodigy Advance, GE Medical Systems) were performed according to the manufacturer guidelines, by trained technicians who inspected the postscan images and made relevant quality corrections to the regions of interest according to a standardized protocol. The DXA device was calibrated each morning with a phantom ahead of measurements. Total body fat in grams and percentage and android fat mass in grams were measured directly by DXA, and VATg and VATcm^3^ were subsequently computed by the validated CoreScan software (EnCore version 17.0). VAT% was calculated as 100 ^*∗*^ VATg divided by android fat mass (g), and subcutaneous fat mass was calculated as android fat mass (g) − VATg. VATindex was calculated as VAT kg/height (m)^2^.

### 2.3. Statistical Analysis

We used STATA 16 (STATA Corp LP, College Station, Texas, USA) to perform all analyses. *P* values were considered significant at a 0.05 level. VATg and VATcm^3^ were highly similar in all analyses; thus, we present only VATg to represent absolute value.

A total of 3675 participants (58.6% women) aged 40–84 years were included (Figure 1). We used descriptive statistics to present characteristics of the study population (Table 1). VAT measures with value of 0 (*n* = 10) were transformed into lowest value (2 g). To compare participants attending only basic examination with those additionally attending extended examinations, we used Student's *t*-test (Supplementary Table 1). We present sex specific means with standard deviations (SDs), confidence intervals (CIs), and percentiles (5^th^, 25^th^, 50^th^, 75^th^, and 95^th^) by 10-year age groups for VATg, VATindex, and VAT% (Supplementary Tables 2–4). To explore the association with VAT (g, index and %) and cardiometabolic risk factors, we used age-adjusted partial Pearson correlation coefficients (Table 2). To investigate the ability of VAT to predict MetS and single MetS components (hypertension, diabetes, elevated triglycerides, and low HDL cholesterol), we performed receiver operating characteristic (ROC) analyses with presentation of age-adjusted area-under-the-curves (AUCs) (Table 3). To further explore if any of the included units of VAT were better than the other in predicting MetS or single MetS components, we used both log likelihood test (Supplementary Table 5) and c-statistics (Supplementary Table 6). As VATg and VATindex were not normally distributed, they were transformed to square root, and z-scores were subsequently created for inclusion to the logistic regression models to study their associations with MetS or single MetS components. In addition, we stratified the analyses of the association between z-scores of VAT (all units) and the presence of MetS or single MetS components in categories of BMI (normal weight, overweight, and obese) (Table 4). Lastly, we used ROC analysis of VAT in prediction of MetS to derive sensitivity and specificity. Further, we applied Youden's index ((sensitivity + specificity)-1) [15] to present suggested threshold values of VAT based on estimated optimal cutoffs (all units) (Table 5) [16]. We used logistic regression analysis to present the odds for MetS for each of the presented threshold values of VAT (all units) (Table 5).

**Figure 2:**
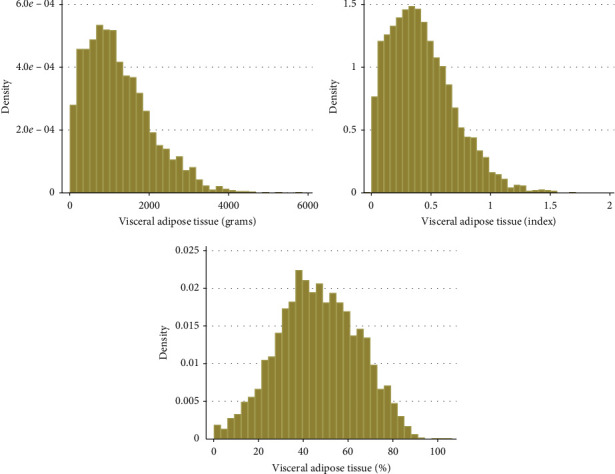
Normality curves of VAT (g), VAT (index), and VAT (%).

**Figure 3:**
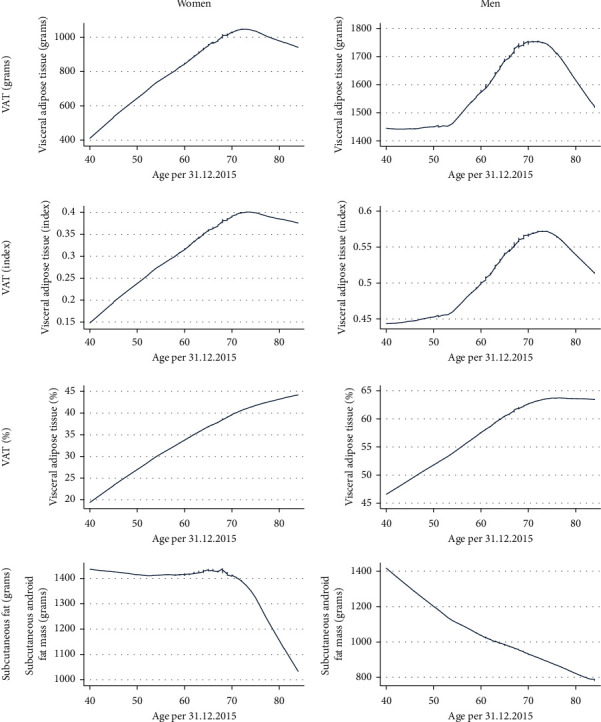
VAT (g, index, and %) and subcutaneous fat (g) in android region over age: the Tromsø Study 2015–2016.

**Figure 4:**
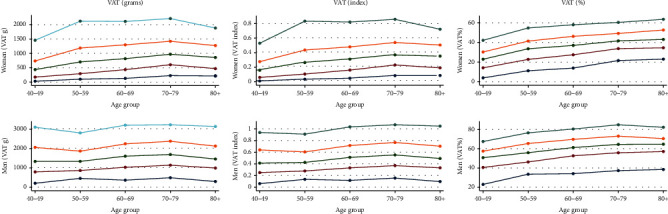
Quantities of VAT (g), VAT (index), and VAT (%) in 10-year age groups in women and men: the Tromsø Study 2015–2016.

Normality distribution of VAT (g, index and %) was checked by visual inspection (Figure 2). VATg and VATindex were positively skewed. We explored how mean VAT (g, index and %) and mean subcutaneous fat change across age (Figure 3). To investigate percentiles (5^th^, 25^th^, 50^th^, 75^th^, and 95^th^) of VAT (g, index and %) over 10-year age groups, we performed line plots with separate lines for each percentile (Figure 4).

## 3. Results

The mean age was 66.2 (8.92) and 65.9 (9.13) years, and mean BMI was 26.8 (4.70) and 27.6 (3.72) kg/m^2^, in women and men, respectively (Table 1). The mean age of the women and men attending only basic examinations was 55.1 (10.9) and 55.9 (11.1). Compared to the participants attending the basic examinations only (Supplementary Table 2), those attending the DXA scanning had lower body weight and height. There were no differences in BMI or waist circumference in women and a small difference in BMI in men (27.9 and 27.6 kg/m^2^, *P*=0.007).

### 3.1. VAT Reference Values

VAT (all measurement units) was higher in men than in women and increased up to age 70 in both genders (Supplementary Tables 2–4 and Figure 3). The exception was VAT%, which continued to increase after attained age of 70–79 in women, while the curve flattened at age 70–79 in men (Figure 3). Subcutaneous fat, however, decreased rapidly after age group 70–79 in women and decreased linearly with age in men, which explains why VAT% continued to increase after age group 70–79, while both VATg and VATindex decreased in women. The investigation of VAT (all measurement units) in percentiles (5^th^, 25^th^, 50^th^, 75^th^, and 95^th^) across age groups showed highly similar patterns for VATg and VATindex in both women and men (Figure 4). In women, the pattern of the 5^th^ percentile (VATg and VATindex) was quite consistent across age, while the 25^th^, 50^th^, and 75^th^ percentile increased until age group 70–79 before it then slightly decreased (Figure 4). The pattern for the 95^th^ percentile (VATg and VATindex) was markedly higher and differed from the other percentiles. In addition, the 95^th^ percentile (VATg and VATindex) in women increased rapidly from age group 40–49 to age group 50–59, remained unchanged until age group 70–79, and decreased thereafter (Figure 4). Percentiles of VAT% in women increased across all age groups, with a steeper increase from age group 40–49 to 50–59 (Figure 4).

In men, the pattern for VATg and VATindex 5^th^ percentile was M-shaped across age groups (Figure 4). In the 25^th^ and 50^th^ percentiles, there was a small continuous increase by age up to age group 70–79, while in the 75^th^ and 95^th^ percentiles, VATg and VATindex decreased from age group 40–49 to 50–59, before it then increased up to age group 70–79 and decreased thereafter. All percentiles for VAT% increased over age groups in men, but the 75^th^ and the 95^th^ percentile decreased after age 70–79 (Figure 4).

### 3.2. VAT and Metabolic Factors

Age-adjusted partial correlation showed that VAT (all measurement units) was positively associated with all cardiometabolic risk factors, except for HDL that was negatively associated with VAT, and total cholesterol that was not significantly associated (Table 2).

When comparing the ability of VATg, VATindex, and VAT% to predict MetS and single MetS components, all AUCs were high (≥0.67). AUCs of VATg and VATindex were consistently higher than AUCs of VAT% (Table 3). When comparing log likelihood/fit of age-adjusted regression models by adding different VAT measurement units to the model with MetS and single MetS components, VATg and VATindex were the most important predictors (Supplementary Table 5). C-statistics revealed no significant differences between predictions of single MetS components from the different VAT measurement units (Supplementary Table 6). In prediction of MetS, there were significant differences by adding both VAT% and VATindex to the model with VATg (*P* ≤ 0.04 for both), but the AUCs were identical in women and only slightly different in men (Supplementary Table 6).

When investigating associations between z-score of VAT (all measurement units) and MetS and single MetS components in categories of BMI, the majority of associations showed significantly higher odds with increasing VAT. The pattern showed highest odds for elevated triglycerides with increasing VAT, in most categories of BMI in women, while the odds for diabetes were highest in most categories of BMI in men (Table 4). With regard to BMI differences, in women the highest odds for hypertension and elevated triglycerides in all z-score units of VAT were observed in the overweight category and the highest odds for diabetes, low HDL cholesterol, and MetS were observed in the obese category. The only exception was z-scores of VATindex in association with low HDL cholesterol which was highest in the overweight category (Table 4). In men, the highest odds for MetS and all MetS components were observed in the overweight category according to all z-score units of VAT (Table 4). The different z-score units of VAT and associations with MetS and MetS components were similar, although higher odds were observed in z-scores of VATg and VATindex compared to VAT%.

Based on Youden's index and the abilities to predict MetS, suggested thresholds of VATg, VATindex, and VAT% in women are ≥1134, ≥0.44, and ≥ 40.3, respectively, with corresponding odds for MetS of 3.63, 4.04, and 3.36, respectively. In men, suggested thresholds and odds for MetS were ≥1859 (OR: 4.03), ≥0.55 (OR: 4.02), and ≥61.2 (OR: 3.12), for VATg, VATindex, and VAT%, respectively (Table 5).

## 4. Discussion

Measures of VAT (expressed in absolute and derived variables) were positively associated with age, MetS, and single MetS components. VATg and VATindex consistently showed slightly stronger associations with MetS and single MetS components than VAT%, but overall, the associations were similar.

Because the distribution of fat, and especially VAT accumulation in abdominal area, rather than overall fat mass has been highlighted as the most robust predictor for cardiometabolic disease, it is valuable to determine reference values for VAT [17]. Relative measures (e.g., waist-to-height ratio) may be stronger predictors for disease than absolute measures (e.g., waist circumference) [18, 19], and it is therefore also relevant to assess whether relative measures of VAT are more strongly correlated with MetS and single MetS components than absolute measures.

We observed higher VATg in percentiles for both women and men as compared to other studies [9, 10]. Miazgowski et al. [9] included young adults only (30–40 years) and Hirsch et al. [10] used the next-generation DXA (iDXA), in addition to presenting different age groups than us (25–49 years and 50+ years), thus limiting direct comparison of results.

Our age-adjusted partial correlations were similar to those found by Rothney et al. [12], who used the iDXA system to study associations between VATg and cardiometabolic risk factors. Miazgowski et al. [9] found stronger correlations between VATg and cardiometabolic risk factors, than in our analysis, but the AUCs observed in the present study are higher. Miazgowski et al. [9] did, however, not adjust for age and presented cardiometabolic risk factors in different units than the present study; thus direct comparison is difficult.

VATg and VATindex decreased rapidly after the age of 70 years. A similar decrease in VAT after age 70 years was found in a study from the United States (using iDXA) [8], which suggested that this decrease could be explained by the small number of participants in the oldest population. This decreasing pattern after age 70 was, however, not confirmed by a larger population-based study (*n* = 10 984) from Ofenheimer et al. [7], conducted in Austria using the system Lunar Prodigy system. Among our participants, 1320 (61% women) were 70 years and older, and together with a high attendance among the older population in the Tromsø Study [20], we conclude that the decrease presented in this study cannot be explained by low participation. Although we did not have the opportunity to investigate potential anthropometric differences in nonattenders, we did compare the population only attending basic examinations, with those also attending extended examinations (DXA) and found no clinically or statistically significant differences in BMI or waist circumference. Previous studies investigating longitudinal change in BMI and waist circumference in the Tromsø Study population showed that adiposity increases with age, with a larger increase in the younger compared to the older birth cohorts [21–23]. One might hypothesize that central adiposity is less prevalent in the older than younger age groups in the present population. Body composition changes with age as total lean mass decreases, fat mass increases, and a greater proportion of fat accumulates within the abdomen as visceral fat [24]. This was reflected in the present study as VAT% (particularly in women) increased continuously, while VATg decreased after age 70. This observation is due to VATg decreasing relatively less than subcutaneous fat. A somewhat surprising finding was the steep decline in subcutaneous fat in women older than 70 years, compared to the linear decrease observed in men.

All cardiometabolic risk factors except total cholesterol showed statistically significant associations with VAT. Similar findings were observed in previous studies investigating the associations between DXA-derived VAT and cardiometabolic risk factors [9, 12, 25]. We could, however, find no other studies comparing how the different measurement units of VAT performed in their associations with MetS and single MetS components. Further, the threshold values were higher for men than women. Although one might expect absolute threshold values to be higher for men, it is quite interesting that the relative measures are also higher, indicating that men might tolerate a higher amount of VAT relative to body size before it becomes a risk for MetS.

To the best of our knowledge, this is the first study presenting reference values for VATindex and VAT% in addition to suggested threshold values for VAT (all units) in association with MetS. Therefore, the results presented are of importance to future studies utilizing DXA-derived VAT and may support clinicians, who use DXA in their daily routine, to make decisions on whether to initialize interventions for patients with abdominal obesity. Although previous studies have presented reference values for VAT in grams, it is highly important to complement previous proposed reference values and also to present reference values for measures that address VAT in relation to overall body size (VATindex and VAT%). It has long been known that VAT is of particular concern to cardiometabolic health, and tracking VAT as a representative for overweight and obesity is a more accurate indicator of the health status than traditional anthropometric measures such as BMI or waist circumference. The current study enables comparison with future studies aiming at presenting reference values and threshold values of VAT. We emphasize that the reference values and thresholds presented are applicable only to similar populations and measures from similar equipment as in the present study. Future studies are needed to confirm the thresholds suggested in our population.

### 4.1. Strengths and Limitations

A major strength of this study is the wide age range of participants, providing proposed VAT reference values in adults aged 40 and older. Further, we could investigate VAT's association with both MetS and single MetS components and propose threshold values for VAT (all units) based on sensitivity and specificity in prediction of MetS. Because of the population-based design, where fasting blood samples are unusual, we did not include fasting glucose as an indicator for diabetes, but rather self-reported diabetes, medication use, or HbA1c ≥ 6.5% which we believe incorporated both diagnosed and undiagnosed diabetes. We did not have the opportunity to examine whether the study participants differed from nonattenders. We did however compare those attending only the basic examination of the survey to those included in this analysis and found no differences according to waist circumference or BMI, and together with the high attendance we conclude that our study is representative of the general population. It should be emphasized that the references derived here (from Lunar Prodigy in age group 40–84 in a Norwegian population) may not be directly comparable to reference values derived from other manufacturers, DXA equipment, age groups, or populations/ethnicities, but to similar populations and methods used in the current study.

## 5. Conclusions

DXA-derived VATg and VATindex increased with age up to 70 years, while VAT% increased continuously with age. The percentiles presented (5^th^, 12^th^, 50^th^, 75^th^, and 95^th^) and suggested thresholds of VATg, VATindex, and VAT% can be used for comparison with studies of similar populations using the same technology. All VAT measurement units except total cholesterol showed statistically significant associations with cardiometabolic risk factors. Results did not substantially differ between measurements units of VAT; thus, any measurement unit seems acceptable to use.

## Figures and Tables

**Table 1:** Descriptive of study population attending dual energy X-ray absorptiometry: the Tromsø Study 2015–2016.

	Women (*n* = 2152)	Men (*n* = 1523)
Age (years (% (*n*))	66.2 (8.92)	65.9 (9.13)
40–49	5.95 (128)	6.70 (102)
50–59	13.2 (284)	12.3 (187)
60–69	43.3 (932)	47.4 (722)
70–79	32.1 (691)	27.5 (418)
80+	5.44 (117)	6.17 (94)
Weight (kg)	71.3 (13.0)	86.0 (13.2)
Height (cm)	163.0 (6.25)	176.4 (6.70)
BMI (kg/m^2^)	26.8 (4.70)	27.6 (3.72)
VAT (g), mean (SD)	936.7 (632.5)	1660.9 (876.6)
VAT (g), median (25p–75p)	832 (444–1302.5)	1578 (1004–2222)
VAT (index), mean (SD)	0.35 (0.24)	0.53 (0.28)
VAT (index), median (25p–75p)	0.32 (0.16–0.49)	0.51 (0.33–0.71)
VAT (%), mean (SD)	37.1 (13.6)	60.2 (14.2)
VAT (%), median (25p–75p)	37.8 (28.3–46.7)	61.2 (52.0–69.9)

Presented as proportion (*n*) or mean (SD). VAT (all measurement units) is also presented as median with 25–75th percentile. VAT index: VAT kg/height (m)^2^.

**Table 2:** Age-adjusted correlation for the association of VATg, VATindex, and VAT% with cardiometabolic risk factors: the Tromsø Study 2015–2016.

Cardiometabolic factors	VATg	VATindex	VAT%
*Women*			
Systolic blood pressure (mmHg)	0.12^*∗*^	0.13^*∗*^	0.10^*∗*^
Diastolic blood pressure (mmHg)	0.08^*∗*^	0.08^*∗*^	0.08^*∗*^
Triglycerides (mmol/L)	0.44^*∗*^	0.44^*∗*^	0.38^*∗*^
Total cholesterol (mmol/L)	−0.006	−0.006	0.01
HDL cholesterol (mmol/L)	−0.43^*∗*^	−0.43^*∗*^	−0.36^*∗*^
HbA1c (%)	0.24^*∗*^	0.24^*∗*^	0.17^*∗*^
CRP (mg/L)	0.14^*∗*^	0.15^*∗*^	0.09^*∗*^
Hypertension	0.20^*∗*^	0.20^*∗*^	0.15^*∗*^
Diabetes	0.23^*∗*^	0.24^*∗*^	0.16^*∗*^
Elevated triglycerides	0.35^*∗*^	0.35^*∗*^	0.31^*∗*^
Low HDL	0.28^*∗*^	0.29^*∗*^	0.24^*∗*^
Metabolic syndrome	0.29^*∗*^	0.30^*∗*^	0.24^*∗*^

*Men*			
Systolic blood pressure (mmHg)	0.11^*∗*^	0.11^*∗*^	0.10^*∗*^
Diastolic blood pressure (mmHg)	0.13^*∗*^	0.11^*∗*^	0.12^*∗*^
Triglycerides (mmol/L)	0.38^*∗*^	0.37^*∗*^	0.31^*∗*^
Total cholesterol (mmol/L)	−0.02	−0.03	−0.02
HDL cholesterol (mmol/L)	−0.38^*∗*^	−0.37^*∗*^	−0.29^*∗*^
HbA1c %	0.27^*∗*^	0.29^*∗*^	0.19^*∗*^
CRP (mg/L)	0.08^*∗*^	0.09^*∗*^	0.03
Hypertension	0.25^*∗*^	0.25^*∗*^	0.21^*∗*^
Diabetes	0.22^*∗*^	0.24^*∗*^	0.18^*∗*^
Elevated triglycerides	0.34^*∗*^	0.34^*∗*^	0.28^*∗*^
Low HDL	0.25^*∗*^	0.26^*∗*^	0.20^*∗*^
Metabolic syndrome	0.30^*∗*^	0.32^*∗*^	0.23^*∗*^

^*∗*^ *P* < 0.001.

**Table 3:** AUC from different age-adjusted models with VAT (g), VAT (%), or VAT (index) in prediction of metabolic syndrome and single metabolic components: the Tromsø Study 2015–2016.

Dependent variables	Women			Men		
	Model 1	Model 2	Model 3	Model 1	Model 2	Model 3
Hypertension	0.77	0.77	0.77	0.73	0.72	0.73
Diabetes	0.72	0.70	0.72	0.75	0.73	0.76
Elevated triglycerides	0.73	0.71	0.73	0.71	0.67	0.71
Low HDL	0.69	0.67	0.69	0.70	0.68	0.70
Metabolic syndrome	0.73	0.71	0.73	0.75	0.73	0.76

Independent: Model 1: age and VATg, Model 2: age and VAT%, Model 3: age and VATindex. Numbers indicating AUC for the model.

**Table 4:** Age-adjusted OR for the association of metabolic syndrome and single metabolic components and standardized/z-scores of VAT mass in categories of body mass index (BMI): the Tromsø Study 2015–2016.

Metabolic factors	zVATg (sqrt)			zVATindex (kg/m^2^(sqrt))			zVAT%		
	Normal weight	BMI overweight	Obese	Normal weight	BMI overweight	Obese	Normal weight	BMI overweight	Obese
*Women*									
Hypertension	1.38 (1.09–1.73)	1.70 (1.32–2.19)	1.30 (0.92–1.84)	1.42 (1.12–1.79)	1.75 (1.35–2.27)	1.34 (0.95–1.90)	1.17 (0.99–1.38)	1.45 (1.19–1.76)	1.23 (0.89–1.71)
Diabetes	1.54 (0.94–2.52)	2.24 (1.47–3.42)	3.68 (2.37–5.70)	1.73 (1.05–2.87)	2.42 (1.56–3.76)	3.60 (2.32–5.60)	1.60 (1.11–2.30)	1.98 (1.42–2.78)	2.10 (1.45–3.02)
Elevated triglycerides	2.37 (1.83–3.06)	2.89 (2.25–3.71)	2.37 (1.75–3.20)	2.50 (1.93–3.24)	3.02 (2.33–3.90)	2.46 (1.80–3.35)	1.64 (1.37–1.97)	2.18 (1.80–2.64)	2.12 (1.60–2.81)
Low HDL	1.94 (1.49–2.52)	2.10 (1.64–2.69)	2.18 (1.62–2.92)	2.05 (1.57–2.67)	2.22 (1.73–2.87)	2.19 (1.63–2.94)	1.55 (1.29–1.87)	1.80 (1.48–2.17)	1.82 (1.40–2.37)
Metabolic syndrome	1.91 (1.40–2.60)	2.30 (1.76–3.02)	2.86 (2.04–4.00)	2.06 (1.50–2.82)	2.46 (1.85–3.25)	2.91 (2.07–4.10)	1.55 (1.24–1.93)	1.91 (1.55–2.36)	2.22 (1.65–2.98)

*Men*									
Hypertension	1.65 (1.22–2.23)	1.97 (1.51–2.56)	1.34 (0.87–2.07)	1.69 (1.25–2.29)	2.09 (1.60–2.74)	1.29 (0.82–2.01)	1.26 (1.03–1.55)	1.64 (1.33–2.01)	1.33 (0.88–2.01)
Diabetes	1.87 (1.02–3.43)	2.99 (1.95–4.59)	2.48 (1.51–4.06)	1.88 (1.03–3.44)	3.51 (2.23–5.51)	2.97 (1.79–4.95)	1.60 (1.03–2.49)	2.50 (1.76–3.55)	1.73 (1.12–2.66)
Elevated triglycerides	1.93 (1.41–2.65)	2.66 (2.09–3.38)	1.73 (1.19–2.52)	1.99 (1.44–2.73)	2.88 (2.25–3.70)	1.71 (1.17–2.50)	1.50 (0.20–1.87)	1.90 (1.58–2.28)	1.25 (0.90–1.74)
Low HDL	1.60 (1.14–2.26)	2.19 (1.71–2.81)	1.47 (1.03–2.09)	1.62 (1.15–2.29)	2.35 (1.81–3.04)	1.59 (1.11–2.28)	1.41 (1.10–1.80)	1.63 (1.34–1.98)	1.30 (0.94–1.78)
Metabolic syndrome	2.13 (1.42–3.20)	2.78 (2.10–3.68)	2.06 (1.40–3.03)	2.18 (1.45–3.27)	3.10 (2.31–4.16)	2.18 (1.47–3.25)	1.63 (1.22–2.18)	1.92 (1.54–2.39)	1.39 (0.99–1.94)

Women: VATg SD: 632.5; VATindex SD: 0.24; VAT% SD: 13.6. Men: VATg SD: 876.6; VATindex SD: 0.28; VAT% SD: 14.2.

**Table 5:** Suggested threshold values of VAT derived from Youden's index and odds for metabolic syndrome: the Tromsø Study 2015–2016.

	Women			Men		
	Threshold	OR (95% CI)	Youden's index	Threshold	OR (95% CI)	Youden's index
VAT (g)	≥1134	3.63 (2.94–4.49)	0.297	≥1859	4.03 (3.17–5.13)	0.331
VAT (index)	≥0.44	4.04 (3.26–4.99)	0.321	≥0.55	4.02 (3.15–5.13)	0.336
VAT (%)	≥40.3	3.36 (2.72–4.15)	0.298	≥61.2	3.12 (2.44–3.97)	0.276

OR: odds ratio; CI: confidence interval.

## Data Availability

No data are publicly available but may be obtained from a third party. The dataset supporting the article findings is available through application directed to the Tromsø Study by following the steps presented on their online page: https://en.uit.no/forskning/forskningsgrupper/sub?p_document_id=453582&sub_id=71247.

## Supplementary Materials

Supplementary Materials Supplementary Table 1: comparison of women and men attending basic examinations and extended examinations in Tromsø 7 (2015–2016). Supplementary Table 2: sex-specific percentiles of VATg by 10-year age groups. Supplementary Table 3: sex-specific percentiles of VATindex by 10-year age groups. Supplementary Table 4: sex-specific percentiles of VAT % by 10-year age groups. Supplementary Table 5: comparison of fit of models (log-likelihood test) by adding VAT % and VATindex to the model with VAT g: the Tromsø Study 2015–2016. Cont. Supplementary Table 5: comparison of fit of models (log-likelihood test) when adding VATg and VATindex to the model with VAT%: the Tromsø Study 2015–2016. Cont. Supplementary Table 5: comparison of fit of models (log-likelihood test) when adding VATg and VAT% to the model with VATindex: the Tromsø Study 2015–2016. Supplementary Table 6: comparison of age-adjusted c-statistics in different models in women: the Tromsø Study 2015–2016. Cont. Supplementary Table 6: comparison of age-adjusted c-statistics in different models in men: the Tromsø Study 2015–2016.
