# Supplementary material for: Reference Values for DXA-Derived Visceral Adipose Tissue in Adults 40 Years and Older from a European Population: The Tromsø Study 2015–2016
Source: J Obes. 2021 May 15;2021:6634536. doi: 10.1155/2021/6634536 (PMC8147540; doi:10.1155/2021/6634536)
Supplement: Supplementary Materials — Supplementary Table 1: comparison of women and men attending basic examinations and extended examinations in Tromsø 7 (2015–2016). Supplementary Table 2: sex-specific percentiles of VATg by 10-year age groups. Supplementary Table 3: sex-specific percentiles of VATindex by 10-year age groups. Supplementary Table 4: sex-specific percentiles of VAT % by 10-year age groups. Supplementary Table 5: comparison of fit of models (log-likelihood test) by adding VAT % and VATindex to the model with VAT g: the Tromsø Study 2015–2016. Cont. Supplementary Table 5: comparison of fit of models (log-likelihood test) when adding VATg and VATindex to the model with VAT%: the Tromsø Study 2015–2016. Cont. Supplementary Table 5: comparison of fit of models (log-likelihood test) when adding VATg and VAT% to the model with VATindex: the Tromsø Study 2015–2016. Supplementary Table 6: comparison of age-adjusted c-statistics in different models in women: the Tromsø Study 2015–2016. Cont. Supplementary Table 6: comparison of age-adjusted c-statistics in different models in men: the Tromsø Study 2015–2016. [file 6634536.f1.docx]

| Supplementary table 1: Comparison of women and men attending basic examinations and extended examinations in Tromsø 7: 2015-2016 | | | | | | |
| --- | --- | --- | --- | --- | --- | --- |
|  | **Women** |  | | **Men** |  |  |
|  | **Basic** | **Extended** | **P-value** | **Basic** | **Extended** | **P-value** |
| N | 8922 | 2152 |  | 8486 | 1523 |  |
| Age (yr (SD)) | 55.1 (10.9) | 66.2 (8.92) | <0.001 | 55.9 (11.1) | 65.9 (9.13) | <0.001 |
| Weight (kg) | 72.9 (14.1) | 71.3 (13.0) | <0.001 | 88.4 (14.3) | 86.0 (13.2) | <0.001 |
| Height (cm) | 164.6 (6.55) | 163.0 (6.25) | <0.001 | 178.0 (6.76) | 176.4 (6.70) | <0.001 |
| BMI (kg/m^2^) | 26.9 (5.00) | 26.8 (4.70) | 0.49 | 27.9 (4.05) | 27.6 (3.71) | 0.007 |
| Waist (cm) | 90.7 (13.0) | 91.2 (12.4) | 0.12 | 100.2 (12.4) | 100.5 (10.5) | 0.32 |
| *BMI; Body mass index | | | | | | |

| Supplementary table 2: Sex specific percentiles of VAT (g) by 10-year age groups | | | | | | | |
| --- | --- | --- | --- | --- | --- | --- | --- |
|  | **Mean (SD)** | **95% CI’s** | **5^th^** | **25^th^** | **50^th^** | **75^th^** | **95^th^** |
| **Women** | 937 (633) | 910 – 963 | 129 | 444 | 832 | 1303 | 2094 |
| **40-49** | 515 (435) | 439 – 591 | 36 | 182 | 437 | 735 | 1455 |
| **50-59** | 832 (651) | 756 – 908 | 102 | 294 | 703 | 1184 | 2044 |
| **60-69** | 935 (636) | 894 – 976 | 131 | 435 | 817 | 1297 | 2104 |
| **70-79** | 1063 (625) | 1016 -1110 | 227 | 606 | 971 | 1421 | 2212 |
| **80+** | 920 (542) | 820 – 1019 | 220 | 477 | 858 | 1267 | 1876 |
| **Men** | 1661 (877) | 1617 – 1705 | 396 | 1004 | 1578 | 2222 | 3158 |
| **40-49** | 1471 (919) | 1291 – 1652 | 215 | 794 | 1335 | 2060 | 3085 |
| **50-59** | 1448 (744) | 1341 - 1556 | 464 | 866 | 1338 | 1871 | 2752 |
| **60-69** | 1687 (891) | 1622 – 1753 | 370 | 1033 | 1607 | 2246 | 3207 |
| **70-79** | 1777 (886) | 1692 – 1862 | 492 | 1141 | 1692 | 2380 | 3226 |
| **80+** | 1568 (823) | 1400 - 1737 | 302 | 995 | 1459 | 2128 | 3118 |

| Supplementary table 3: Sex specific percentiles of VAT index by 10-year age groups | | | | | | | |
| --- | --- | --- | --- | --- | --- | --- | --- |
|  | **Mean (SD)** | **95% CI’s** | **5^th^** | **25^th^** | **50^th^** | **75^th^** | **95^th^** |
| **Women** | 0.35 (0.24) | 0.34 – 0.36 | 0.05 | 0.16 | 0.32 | 0.49 | 0.80 |
| **40-49** | 0.19 (0.16) | 0.16 – 0.21 | 0.01 | 0.06 | 0.16 | 0.27 | 0.49 |
| **50-59** | 0.31 (0.24) | 0.28 – 0.34 | 0.03 | 0.11 | 0.26 | 0.43 | 0.80 |
| **60-69** | 0.35 (0.24) | 0.34 – 0.37 | 0.05 | 0.16 | 0.31 | 0.48 | 0.82 |
| **70-79** | 0.41 (0.24) | 0.39 – 0.42 | 0.09 | 0.23 | 0.37 | 0.54 | 0.85 |
| **80+** | 0.36 (0.21) | 0.33 – 0.40 | 0.09 | 0.19 | 0.35 | 0.51 | 0.72 |
| **Men** | 0.53 (0.28) | 0.52 – 0.55 | 0.12 | 0.33 | 0.51 | 0.71 | 1.02 |
| **40-49** | 0.45 (0.28) | 0.40 – 0.51 | 0.07 | 0.25 | 0.41 | 0.64 | 0.93 |
| **50-59** | 0.46 (0.24) | 0.43 – 0.49 | 0.14 | 0.28 | 0.43 | 0.61 | 0.88 |
| **60-69** | 0.54 (0.28) | 0.52 – 0.56 | 0.12 | 0.33 | 0.51 | 0.71 | 1.02 |
| **70-79** | 0.58 (0.29) | 0.55 – 0.61 | 0.16 | 0.37 | 0.55 | 0.77 | 1.07 |
| **80+** | 0.52 (0.27) | 0.47 – 0.58 | 0.10 | 0.34 | 0.49 | 0.70 | 1.05 |

| Supplementary table 4: Sex specific percentiles of VAT % by 10-year age groups | | | | | | | |
| --- | --- | --- | --- | --- | --- | --- | --- |
|  | **Mean (SD)** | **95% CI’s** | **5^th^** | **25^th^** | **50^th^** | **75^th^** | **95^th^** |
| **Women** | 37.1 (13.6) | 36.5 - 37.7 | 13.0 | 28.3 | 37.8 | 46.7 | 58.8 |
| **40-49** | 22.7 (11.1) | 20.8 - 24.7 | 4.35 | 14.2 | 22.9 | 30.2 | 41.6 |
| **50-59** | 32.7 (13.2) | 31.1 - 34.2 | 11.4 | 22.9 | 33.6 | 41.4 | 54.6 |
| **60-69** | 36.5 (13.5) | 35.6 - 37.4 | 14.0 | 27.5 | 37.0 | 46.2 | 57.9 |
| **70-79** | 41.3 (11.8) | 40.5 - 42.2 | 21.6 | 33.7 | 41.6 | 49.2 | 60.4 |
| **80+** | 43.1 (12.8) | 40.7 - 45.4 | 23.2 | 34.6 | 43.1 | 52.7 | 63.5 |
| **Men** | 60.2 (14.2) | 59.4 - 60.9 | 34.3 | 52.0 | 61.2 | 69.9 | 80.9 |
| **40-49** | 48.7 (14.3) | 45.9 - 51.5 | 24.2 | 40.6 | 50.8 | 57.5 | 67.6 |
| **50-59** | 55.5 (13.0) | 53.6 - 57.4 | 33.6 | 46.3 | 55.9 | 65.5 | 76.0 |
| **60-69** | 60.5 (13.7) | 59.5 - 61.4 | 34.4 | 52.7 | 61.3 | 69.8 | 80.3 |
| **70-79** | 63.9 (14.0) | 62.5 - 65.2 | 37.3 | 55.9 | 64.6 | 73.2 | 85.0 |
| **80+** | 63.0 (13.2) | 60.3 - 65.7 | 39.3 | 57.4 | 64.8 | 70.6 | 82.4 |

| Supplementary table 5: Comparison of fit of models (log likelihood test) by adding VAT % and VATindex to the model with VATg: the Tromsø Study 2015-2016 | | | | |
| --- | --- | --- | --- | --- |
| **Dependent variables** | **Women** |  | **Men** |  |
|  | **P-value (M1 vs. M2)** | **P-value (M2 vs. M3)** | **P-value (M1 vs. M2)** | **P-value (M2 vs. M3)** |
| Hypertension | 0.90 | 0.03 | 0.22 | 0.20 |
| Diabetes | 0.37 | 0.14 | 0.009 | 0.001 |
| Elevated triglycerides | <0.001 | 0.04 | 0.08 | 0.06 |
| Low HDL | 0.006 | 0.07 | 0.12 | 0.01 |
| Metabolic syndrome | 0.006 | 0.02 | 0.11 | 0.008 |
| Independent: Model 1: age and VATg, Model 2: age, VATg and VAT%, Model 3: age, VATg, VAT% and VATindex  *p-value indicating whether adding variables significantly improve the fit of the model. | | | | |

| Cont. Supplementary table 5: Comparison of fit of models (log likelihood test) when adding VATg and VATindex to the model with VAT%: the Tromsø Study 2015-2016 | | | | |
| --- | --- | --- | --- | --- |
| **Dependent variables** | **Women** |  | **Men** |  |
|  | **P-value (M1 vs. M2)** | **P-value (M2 vs. M3)** | **P-value (M1 vs. M2)** | **P-value (M2 vs. M3)** |
| Hypertension | <0.001 | 0.03 | <0.001 | 0.20 |
| Diabetes | <0.001 | 0.14 | <0.001 | 0.001 |
| Elevated triglycerides | <0.001 | 0.04 | <0.001 | 0.06 |
| Low HDL | <0.001 | 0.71 | <0.001 | 0.01 |
| Metabolic syndrome | <0.001 | 0.02 | <0.001 | 0.008 |
| Independent: Model 1: age and VAT%, Model 2: age, VAT% and VATg, Model 3: age, VAT%, VATg and VATindex  *p-value indicating whether adding variables significantly improve the fit of the model. | | | | |

| Cont. Supplementary table 5: Comparison of fit of models (log likelihood test) when adding VATg and VAT% to the model with VATindex: the Tromsø Study 2015-2016 | | | | |
| --- | --- | --- | --- | --- |
| **Dependent variables** | **Women** |  | **Men** |  |
|  | **P-value (M1 vs. M2)** | **P-value (M2 vs. M3)** | **P-value (M1 vs. M2)** | **P-value (M2 vs. M3)** |
| Hypertension | 0.30 | 0.69 | 0.98 | 0.35 |
| Diabetes | 0.99 | 0.42 | 0.04 | 0.05 |
| Elevated triglycerides | 0.81 | 0.002 | 0.97 | 0.19 |
| Low HDL | 0.76 | 0.01 | 0.26 | 0.30 |
| Metabolic syndrome | 0.46 | 0.01 | 0.41 | 0.31 |
| Independent: Model 1: age and VATindex, Model 2: age, VATindex and VATg, Model 3: age, VATindex, VATg and VAT%  *p-value indicating whether adding variables significantly improve the fit of the model. | | | | |

| Supplementary table 6: Comparison of age adjusted c-statistics in different models in women: The Tromsø Study 2015-2016 | | | | | | | |
| --- | --- | --- | --- | --- | --- | --- | --- |
| **Dependent variables** | **Women** |  |  |  |  |  |  |
|  | **AUC (M1)** | **AUC (M2)** | **AUC (M3)** | **P-value (M1 vs. M2)** | **P-value (M1 vs. M3)** | **P-value (M2 vs. M3)** |  |
| Hypertension | 0.77 | 0.77 | 0.78 | 0.80 | 0.12 | 0.12 |  |
| Diabetes | 0.72 | 0.72 | 0.73 | 0.11 | 0.06 | 0.20 |  |
| Elevated triglycerides | 0.73 | 0.73 | 0.73 | 0.68 | 0.22 | 0.08 |  |
| Low HDL | 0.69 | 0.69 | 0.70 | 0.49 | 0.13 | 0.07 |  |
| Metabolic syndrome | 0.73 | 0.73 | 0.73 | 0.25 | 0.02 | 0.02 |  |
| Independent: Model 1: age and VATg, Model 2: age, VATg and VAT%, Model 3: age, VATg, VAT% and VATindex  *Numbers indicating AUC for the model, p value presents whether there is a significant AUC difference between the models | | | | | | | |

| Cont. supplementary table 6: Comparison of age adjusted c-statistics in different models in men: The Tromsø Study 2015-2016 | | | | | | | |
| --- | --- | --- | --- | --- | --- | --- | --- |
| **Dependent variables** | **Men** |  |  |  |  |  |  |
|  | **AUC (M1)** | **AUC (M2)** | **AUC (M3)** | **P-value (M1 vs. M2)** | **P-value (M1 vs. M3)** | **P-value (M2 vs. M3)** |  |
| Hypertension | 0.73 | 0.73 | 0.73 | 0.50 | 0.17 | 0.23 |  |
| Diabetes | 0.75 | 0.76 | 0.77 | 0.37 | 0.10 | 0.18 |  |
| Elevated triglycerides | 0.71 | 0.71 | 0.71 | 0.86 | 0.23 | 0.07 |  |
| Low HDL | 0.70 | 0.70 | 0.71 | 0.32 | 0.02 | 0.03 |  |
| Metabolic syndrome | 0.75 | 0.75 | 0.76 | 0.31 | 0.02 | 0.04 |  |
| Independent: Model 1: age and VATg, Model 2: age, VATg and VAT%, Model 3: age, VATg, VAT% and VATindex  *Numbers indicating AUC for the model, p value presents whether there is a significant AUC difference between the models | | | | | | | |
